# Authors’ Reply to: Screening Tools: Their Intended Audiences and Purposes. Comment on “Diagnostic Accuracy of Web-Based COVID-19 Symptom Checkers: Comparison Study”

**DOI:** 10.2196/26543

**Published:** 2021-05-21

**Authors:** Nicolas Munsch, Alistair Martin, Stefanie Gruarin, Jama Nateqi, Isselmou Abdarahmane, Rafael Weingartner-Ortner, Bernhard Knapp

**Affiliations:** 1 Data Science Department Symptoma Vienna Austria; 2 Medical Department Symptoma Attersee Austria; 3 Department of Internal Medicine Paracelsus Medical University Salzburg Austria; 4 Department of Computer Science University of Applied Sciences – Technikum Wien Vienna Austria

**Keywords:** COVID-19, symptom checkers, benchmark, digital health, symptom, chatbot, accuracy

We thank Millen et al [1] (Ada Health) for having taken the time to read our recently published COVID-19 symptom checker comparison study [2] and for their letter to the editor. Millen et al [1] identified three opportunities for improvement to our manuscript. We wish to provide an itemized response to their letter as outlined below.

First, Millen et al [1] state that the Symptoma symptom checker is a fundamentally different tool, which “might be useful in some hospital settings as a clinical decision support tool“ but should not be compared to other layperson COVID-19 symptom checkers. We disagree with this assessment. Indeed, the opposite is true: the same Symptoma engine is widely used by laypersons and therefore the comparison as executed in our publication is appropriate.

Second, Millen et al [1] state that Symptoma’s superior accuracy is due to its unique ability “to make use of professional interpretation of clinical findings” and that such data should not be used by any of the symptom checkers. At Symptoma, we have found that the greater the wealth of data flowing into the symptom checker AI (artificial intelligence), the better the output and results generated for our users. Further, Millen et al [1] state that “Symptoma does not indeed perform superiorly” if no clinical findings are taken into account while referring to the appendix of our study [2]. We respectfully disagree with this on several fronts: (1) the appendix shows a different analysis than that referred to by Millen et al [1] and (2) that if the analysis is modified to fit Millen et al’s [1] suggestion, which we consider less appropriate, the ranking still remains unchanged. The F1 scores will be slightly modified (from 0.92 to 0.90 for “high risk” only while it remains at 0.91 for "high risk" and "medium risk") while still ranking Symptoma first.

Lastly, Millen et al [1] suggest that the evaluation of their symptom checker should not be based on “accuracy.” The most prevalent outcome parameter of comparative symptom checker studies is diagnostic accuracy [3-5]. Even though the symptom checker results do not represent a diagnosis, the importance of accurate results as generated by a symptom checker is paramount to its functionality. Other outcome parameters such as the ones suggested by Millen et al [1] are poorly measurable, harbor the potential for bias, and lack comparability.

## Abbreviations

AI: artificial intelligence

## References

[1] Millen E, Gilsdorf A, Fenech M, Gilbert S (2021). Screening Tools: Their Intended Audiences and Purposes. Comment on “Diagnostic Accuracy of Web-Based COVID-19 Symptom Checkers: Comparison Study”. J Med Internet Res.

[2] Munsch N, Martin A, Gruarin S, Nateqi J, Abdarahmane I, Weingartner-Ortner R, Knapp B (2020). Diagnostic Accuracy of Web-Based COVID-19 Symptom Checkers: Comparison Study. J Med Internet Res.

[3] Semigran HL, Linder JA, Gidengil C, Mehrotra A (2015). Evaluation of symptom checkers for self diagnosis and triage: audit study. BMJ.

[4] Nateqi J, Lin S, Krobath H, Gruarin S, Lutz T, Dvorak T, Gruschina A, Ortner R (2019). [From symptom to diagnosis-symptom checkers re-evaluated : Are symptom checkers finally sufficient and accurate to use? An update from the ENT perspective]. HNO.

[5] Semigran Hannah L, Levine David M, Nundy Shantanu, Mehrotra Ateev (2016). Comparison of Physician and Computer Diagnostic Accuracy. JAMA Intern Med.

